# An outbreak of appreciation: A discursive analysis of tweets of gratitude expressed to the National Health Service at the outset of the COVID‐19 pandemic

**DOI:** 10.1111/hex.13359

**Published:** 2021-09-20

**Authors:** Giskin Day, Glenn Robert, Kathleen Leedham‐Green, Anne Marie Rafferty

**Affiliations:** ^1^ Division of Methodologies Research, Florence Nightingale Faculty of Nursing, Midwifery and Palliative Care King's College London London UK; ^2^ Medical Education Research Unit, School of Medicine Imperial College London London UK

**Keywords:** COVID‐19, discursive psychology, gratitude, NHS, Twitter

## Abstract

**Background:**

The early stages of the coronavirus disease 2019 pandemic prompted unprecedented displays of gratitude to healthcare workers. In the United Kingdom, gratitude was a hotly debated topic in public discourse, catalysing compelling displays of civic togetherness but also attracting criticism for being an unhelpful distraction that authorized unrealistic expectations of healthcare workers. Expressions of thanks tend to be neglected as drivers of transformation, and yet, they are important indicators of qualities to which people attach significance.

**Objective:**

This study aimed to use discursive analysis to explore how the National Health Service (NHS) was constructed in attention‐attracting tweets that expressed and/or discussed gratitude to the NHS.

**Methods:**

Having determined that Twitter was the most active site for traffic relating to gratitude and the NHS, we established a corpus of 834 most‐liked tweets, purposively sampled from Twitter searches on a day‐by‐day basis over the period of the first lockdown in the United Kingdom (22 March–28 May 2020). We developed a typology for tweets engaging with gratitude as well as analysing what the NHS was thanked for.

**Results:**

Our analysis, informed by a discursive psychology approach, found that the meanings attributed to gratitude were highly mobile and there were distinct patterns of activity. The NHS was predominantly—and sometimes idealistically—thanked for working, effort, saving and caring. Displays of gratitude were seen as incommensurable with failures of responsibility. The clap‐for‐carers campaign was a potent driver of affect, especially in the early parts of the lockdown.

**Conclusions:**

The social value of gratitude is implicated in the re‐evaluation of the risks and rewards of healthcare and social care work in the wake of the pandemic. We caution against cynicism about gratitude overshadowing the well‐being effects that expressing and receiving gratitude can engender, particularly given concerns over the detrimental effects of the pandemic on mental health.

**Public Contribution:**

This study involves the analysis of data provided by the public and published on social media.

## 1 INTRODUCTION

The coronavirus disease 2019 (COVID‐19) pandemic is likely to present the highest collective level of biopsychosocial threat for a generation. While the early stages of the pandemic were characterized by mostly negative emotions, like fear and anxiety,^1^ it will also be remembered in the United Kingdom for the conspicuous displays of gratitude to the National Health Service (NHS), healthcare workers and other key workers. Much has been written about the misalignment of gratitude rhetoric and material conditions for healthcare workers, from insufficient supplies of personal protective equipment (PPE) at the start of the pandemic, to problems with testing availability, to the much‐derided 1% pay rise offer for NHS staff in England.^2^, ^3^ Yet, instead of dismissing displays of gratitude as tokenistic, superficial and sentimental, it is worth considering what we can usefully learn from them. In healthcare generally, there tends to be a disproportionate emphasis on relying on learning from what people complain about rather than what they appreciate.^4^, ^5^ However, unprompted expressions of gratitude are important indicators of what people value. A recent study on person‐centred care shows that Twitter is a useful source of public data on expectations of healthcare.^6^ As healthcare is reconfigured in the wake of the pandemic, the NHS needs to be recognized as an arena for cultural and social practices rather than merely an institution in need of organisational change. Paying attention to the qualities to which people attach significance could inform transformation through the lens of appreciation rather than criticism.

Gratitude as a personal, social and health benefit has grown in prominence during the pandemic. Consistent with past research that has shown that gratitude motivates prosocial behaviour,^7^ studies focusing on COVID‐19 found that participants who were grateful or thankful were more willing to endorse measures that helped curtail the spread of the virus.^8^, ^9^ Practising gratitude has been implicated as a predictor of well‐being during lockdown.^10^ Gratitude journaling is recommended in many of the online well‐being courses that have proliferated during lockdowns, including the ‘Science of Happiness’ free online course that has attracted over 3 million enrolments.^11^ There is a growing body of research that supports Fredrickson's ‘broaden and build’ model, in which experiencing gratitude, along with other positively valenced emotions, *broadens* the repertoires of action that people are prepared to take.^12^ Immediate effects sparked by positive emotion tend to be relatively short‐lived, but the model predicts that these actions *build* durable resources that can be drawn on as coping strategies to survive and thrive.

Our recent meta‐narrative review of gratitude in healthcare identified a need for research on the ways in which gratitude acquires meaning in real‐world situations.^13^ Yoshimura and Berzins^14^ have called for a focus on expressions of gratitude to extend and enrich the plethora of research on gratitude experiences. In particular, they call for examinations of the semantic features of gratitude expressions and the topics on which people focus when thanking others. Our study addresses this study gap by exploring the features of expressions of gratitude associated with the NHS on Twitter, and how the volume and nature of these expressions changed over the course of the first lockdown in the United Kingdom (22 March–28 May 2020).

Because our phenomenon of interest is the social expression of gratitude, we have elected not to focus on the online identities of tweeters or the networks that they inhabit. Offline and online identities are fluid, particularly during times of crisis: politicians become patients, scientists become celebrities and citizens become campaigners. Motivations for tweeting about gratitude may have included impression management and identity positioning. However, rather than speculate on those identities and motives, we chose to characterize the content, function and form of popular tweets, exploring how these modulated over time and in response to events.

This study is also an investigation into the potential for social media data to be explored using an approach informed by discursive psychology. It adds to the relatively few studies that have used this approach to explore tweets (e.g., Hurst^15^ and Rasmussen^16^). Unlike the everyday conversational routines that usually comprise the data source for discursive psychology, exchanges on Twitter are asynchronous and constrained by the features for interaction afforded by the platform. Tweets do, however, perform the types of social and psychological actions that are paradigmatic to discursive psychology, and the methodology has been proposed as having the potential to play a very important role in understanding online interactions.^17^

Discursive psychology is a distinct branch of discourse analysis that looks at talk with respect to what it *does* rather than what it *reflects*.^18^ The central concern of discursive psychology is how psychological characteristics are handled as part of participants' practices and orientations, performed as social action in how they talk^19^—or, in this case, how they tweet. This is not to discount the alignment of felt emotions with what people say, but a discursive psychology approach does not assume that what is said is a transparent relay to underlying states of mind. This approach gains support from recent experimental research in psychobiology that challenges notions of ‘basic’ natural emotions, arguing instead that emotions are ‘made’ and any category of emotion is filled with variety^20^—a stance that we consider to be particularly applicable to a complex emotion like gratitude.

Hitherto, the dominant paradigm for investigating the relationship between language and emotions has been cognitive psychology. Cognitive approaches treat language as referring to or representing ‘inner states’: There is an assumption that there is a reality behind the talk that language allows us to access.^17^ For example, Kleinberg et al.^21^ assembled a ‘ground truth data set’ of emotional responses to the COVID‐19 pandemic, arguing that the core aim of emotion detection is to ‘make an inference about the author's emotional state’. Rather than investigating whether a tweet is written in a pessimistic tone, they are interested in whether the author of the tweet *actually felt* pessimistic. While this aim is admirable, it is predicated on the questionable assumption that constructed texts are direct relays to people's emotions. In an excoriating critique of a study by Mitchell et al.^22^ that used Twitter to map ‘the geography of happiness’, Jensen^23^ has cogently outlined the dangers of conflating online social life with offline emotional states, along with other limitations of this type of research, such as sampling bias and overextending inferences.

To sum up, our research is couched in an ontology of the relational encounter: It starts from the point of view that gratitude takes place within the context of acts of communication. Underpinning our approach is a constructivist perspective, which assumes that social actors are in a continuous process of reinvention. Therefore, we do not assume a fixed identity for tweeters and eschew simplistic categorisations based on biographies. Here, gratitude is investigated as a *discursive practice*: It is strategized as purposeful, performative action, a social and cultural resource upon which actors draw. This approach rejects an epistemology of thanking expressions as the explicit representation of an implicit emotion and embraces one in which textual, verbal, visual and gestural actions participate in the pragmatic construction of the objects and objectives of language.

## 2 MATERIALS AND METHODS

### 2.1 Characterizing and compiling the data set

To inform our choice of the multiple available social media platforms on which to focus our research, we used the social media listening service Brand24 (brand24.com) to monitor traffic relating to gratitude and the NHS. Twitter was found to be by far the most active site for mentions of NHS AND (gratitude OR grateful OR thank OR thanks). Twitter is a dynamic platform, and its affordances and features are often updated. At the time of data collection, Twitter is a publicly available platform that offers free accounts to those who wish to tweet or to follow (or subscribe to) specific accounts, but no account is needed to access or search the site. Tweet entry is limited to 280 characters. Tweeters can add up to four photographs, a graphic and short bursts of video. Twitter users can respond to a tweet by commenting on it, ‘liking’ it, quoting it and/or retweeting it. The visibility of tweets depends on the privacy settings selected by the tweeter, Twitter's proprietary algorithms that personalize what appears on users' news feeds and whether advertisers have paid for a tweet to be promoted.

A dictionary definition of gratitude is relatively uncontroversial: ‘The quality or condition of being grateful; a warm sense of appreciation of kindness received, involving a feeling of goodwill towards the benefactor and a desire to do something in return’.^24^ However, the nuanced meanings of gratitude, the appropriate application of the term and the characterisation of its value are highly contested.^25^ Gratitude has been found to be prototypically organized: Features of gratitude do not belong to classically defined categories, the membership of which is specified by in/out criteria. Instead, gratitude is a concept made up of a ‘fuzzy collection of features’, some of which are considered more central than others.^26^ Our rationale for searching for tweets prioritized relevance to the phenomenon of interest (gratitude in relation to the NHS) rather than representativeness or comprehensiveness. We concentrated on the two features ranked as being most central to gratitude by UK participants in a prototype analysis^27^: ‘thankful’ and ‘grateful’. We searched for linguistic variants of these using the search string ‘NHS AND (gratitude OR grateful OR thank OR thanks OR #thank) lang:EN min_faves:300 until:[day after date of interest] since:[date of interest]’.

Twitter is opaque about when it starts to impose its own filters or limits numbers of search results (its guidelines state that it filters for ‘quality tweets and accounts’).^28^ To reduce the chance of search results being capped, the volume of returns was limited by running the search separately for each day from 1 March to 23 June 2020. Search returns were manually sifted for relevance to the research question (‘Does this tweet engage with issues of gratitude to the NHS?’). Tweets in which gratitude was not addressed to the NHS (e.g., ‘Thank you everyone for staying home to protect our NHS’) constituted about 30% of the search results and were excluded.

The search was focused to capture a flavour of issues gaining traction on Twitter by filtering for ‘likes’—an admittedly crude measure of salience (for a roundup of the complicated politics of ‘liking’ see Taylor),^29^ but an instrumentally useful one for harvesting higher‐impact tweets. We acknowledge that the impetus to ‘like’ a tweet is not necessarily based on agreeing with its content—‘liking’ may be indiscriminate or based on the status of the tweeter—but the amplification of certain messages became part of the online phenomenon itself. Regardless of the triggers for their amplification, Twitter users saw and engaged with the tweets in our data set. A threshold of 300 ‘likes’ allowed for a manageable number of tweets to be returned (range: 8–55, median: 22.4 after sifting for relevance every day in the first 2 weeks of the sampling period). The search was run at least 1 week after the date of interest to allow the number of ‘likes’ to accumulate. Details of each tweet were drawn into an Excel spreadsheet and imported into NVivo for coding.

### 2.2 Constructing the coding frame

To construct the initial coding frame, we selected a stratified sample of 100 tweets from across the sampling period for inductive coding, leading to a list of characteristics useful for describing the tweet. This inductive approach is consistent with the ‘emic’ focus of discursive psychology,^17^ in which we worked with the categories that we recognized in the corpus rather than imposing preconceived categories. We approached tweets as ‘micronarratives’ consisting of characters, actions, objects, contexts and instruments.^30^ Informed by Haugh's study of im/politeness as social practice,^31^ our coding focused on the action component or *function* of each tweet (what was it doing?) and the *plot* of the tweet—what was it about? Once a draft codebook had been agreed, a second sample of tweets was assembled, coded independently and discussed until consensus was reached. The codebook was further refined during comparison of results; for example, we merged the codes of ‘instructing/directing’ and ‘requesting/asking’ because coders had difficulty distinguishing between the two. The resulting typology of gratitude that we used to code our data set is shown, with examples, in Table 1.

**Table 1:** Typology of tweets of gratitude

	Code	Description	Examples (paraphrased)	No. of tweets coded
Function (what the tweet does)	Commemorating	Words of gratitude in relation to deaths	Our Filipino comrades who worked in the NHS and social care and unfortunately and very sadly died due to the pandemic. RIP [Folded hands emoji]. Thank you for all your service [collage of photos]. [Named individual] came out of retirement after a long NHS career to help fight the pandemic. He lost his life saving others. Rest easy hero. Thank you for your bravery [Broken heart emoji]	31
	Commenting, critiquing or criticizing	Commentary on the nature of gratitude or an issue associated with gratitude	This campaign isn't just about saying thank you now in the midst of COVID‐19. It's about recognising everyone who works around the clock to keep the NHS going even when there isn't a pandemic happening. Thank you NHS When this is all over, I really hope we find a suitable way to thank the doctors and nurses who have come out of retirement to fight the pandemic, and all the NHS workers who are extremely special	114
	Describing or sharing news	An announcement or a statement of news, including links to news articles	[Penguin emoji] [Blue heart emoji] NHS PENGUIN CHICKS NAMED! These penguin chicks have been named after NHS heroes and hospitals. Our staff will make sure they're fully cared for, just like the NHS care for us every day. A thank you to all of our NHS heroes [video of penguin chicks being weighed] So proud to reveal this amazing piece artwork created by #Banksy as a thank you to all those who work with and for the NHS and [named] hospital. An inspirational backdrop to pause and reflect in these unprecedented times. [photograph of artwork]	407
	Instructing or requesting	Instructions, requests, pleas, directions or invitations	Paramedics came and took my partner to hospital today. He's been unwell for a while and wasn't getting better. Thank you #NHS for continuing your work and helping to save lives. Can all the idiots that don't think this is real stop going outside now please? Footfall in the city centre is down 90%. Thank you to everyone who's staying at home. Please stay 2 m apart when you go out. Thank you to the NHS, social care & essential workers who are out saving lives. Respect them. #StayHomeSaveLives	144
	Reacting	This is a response to receiving or witnessing an act of gratitude	Thank you to the Queen for speaking for us all tonight in your thanks to NHS and key workers, for giving us confidence in our national virtues and also hope in these dark times. I am in absolute hysterics over this, it is the strangest supportive gesture I've ever seen. Can you imagine any NHS worker seeing this and thinking ‘thank you I feel supported’? [quotes tweet featuring video of ferry of London's Woolwich Ferry performing ‘doughnuts' on the Thames]	154
	Recognizing	Enactment of gratitude. Words or performance	Thank you to @NHSuk and all the medical staff around the world [red heart emoji] Nearly 3 weeks in hospital, nearly died, but today [named person] came home [Folded hands emoji]. Thought this day would never come. Thank you to our incredible NHS #COVID19 #NHSThankYou	638
	Signalling values	Drawing attention to personal or professional values	Our country is going to be tested. But I know that, if we emulate the selflessness, compassion and commitment of our outstanding NHS staff, police, firefighters and emergency workers, there is nothing that we cannot overcome. Thank you [praying hands emoji] Ramadan Mubarak to everyone welcoming in the month of Ramadan. I want to pay tribute to all the Muslims working in our NHS, our care service and elsewhere on the front line of our fight against coronavirus. Thank you for keeping us safe. [video message]	29
	Benefit	Refers to a benefit offered in thanks, usually as a perk	Well done [named branch of a supermarket]. Well organised entry, checking NHS identities, fairly well stocked of the essentials and beautiful flowers to thank me for working for our NHS. Thank you I've closed my holiday house but I have the absolute pleasure of giving the keys to a local NHS worker who doesn't want to risk taking the virus back to vulnerable family members. Thank you to our amazing NHS staff and key workers	68
Plot (what the tweet is about)	Fundraising	Initiatives to raise money in gratitude	PE teacher Joe Wicks has raised £200,000 for NHS Charities Together fund through his online classes, in gratitude to the medical staff following his hand surgery We're thrilled to reveal our new away kit which is available to preorder now. Like the home shirt, it conveys our thanks to the frontline heroes of the NHS and is part of our wider fundraising efforts for them	38
	Performance	Creative action, for example videos, drawings, banners, buildings lit in blue	[Rainbow emoji] Rainbows have become a symbol of hope and the NHS during the current pandemic, so we thought what better way to show our thanks to our amazing NHS and key workers, than to re‐brand our bus to a rainbow NHS bus? [photo of bus] Thank you NHS [Clapping hands emoji] [Thumbs up emoji] We're showing our support to the incredible NHS workers who are working tirelessly to help those affected by the pandemic by decorating a number of our postboxes. The postboxes are painted in NHS blue and say ‘Thank You NHS’. [photographs of postbox]	291
	Political, social or economic	Comments on political, social or economic factors in relation to gratitude	Thanks for the clapping but after a decade of voting for a party who always stripped the NHS and tried to sell it off, it is a bit of an empty gesture. Please vote in the future for a party who supports the NHS if you mean that clap seriously The prime minister's nurses Luis (from Portugal) and Jenny (from New Zealand) now have their measure of his gratitude: confirmed that, on top of the taxes they already pay, they will have to pay the NHS migrant tax	100
	Social culture	Substantive comments on behavioural or social compliance or solidarity.	[Loudspeaker emoji] #ClapForCarers is happening again tonight at 8 pm. Let's join together to say a huge thank you to all NHS staff, carers and key workers [clapping hands emoji] #ClapForNHS #ClapForCarers #ThankYouNHS I beg you, do not release sky lanterns as way of saying thank you to NHS workers. They are dangerous to people, wildlife and for the environment as a whole. Clapping is perfect. I haven't met an NHS worker yet who wants this [quote of tweet advocating releasing lanterns in support of NHS]	64
	Specific act	Specifies an action that is gratitude‐worthy	I want to put this on the record: thank you to everyone at [named hospitals] and all in the @NHSuk for working during Easter break. The sacrifices you are making to protect us are incomprehensible. Congratulations to final year medical students who graduated early this week. They will help @NHSuk respond to the extraordinary challenges of the pandemic. We owe you and everyone in the #NHS a huge thank you and wish you well. #ThankYouNHS	82
	Treatment or care experience	Personal experiences with connection to treatment or care	Two months ago my aunt was admitted into hospital with coronavirus. Our family was told to prepare for the worst. Today she was applauded by NHS staff as she left the hospital. As a family, you will forever be in our gratitude and prayers #NHSheroes Giving birth to twins prematurely during this pandemic and staying on the ward for a week has made me so grateful for the littlest things. Forever in debt to the NHS for doing their best for my babies	134
	Words of appreciation (words themselves enact the gratitude)	Expressions of thanks	To everyone working hard for our communities and vital services—the NHS heroes and others: THANK YOU The Prime Minister has thanked doctors and nurses for the ‘exemplary’ care he received. ‘I can't thank them enough. I owe them my life’	634

Abbreviation: NHS, National Health Service.

### 2.3 Coding the data

For the full data set of 834 tweets, we followed the principles and protocols for consensual qualitative research–modified.^32^, ^33^ Coding was additive: Each tweet was coded for at least one function and one plot, but as many codes as were relevant were applied. GD coded all the tweets, GR coded 60% and KLG coded 40% so that each tweet was coded independently by two coders before they were discussed and coding agreed. AMR audited the coding. GD and KLG narratively coded for metaphors in tweets. Additionally, in vivo coding was used to capture explicit mentions of what the NHS was being thanked for and references to groups or individuals to whom the thanks was being addressed.

### 2.4 Ethical considerations

Although Twitter is a public platform and this study does not include sensitive personal information, private people may have an expectation that their tweets are specific to the context of Twitter rather than being the subject of research. We have drawn on recommended frameworks for ethical use of social media in research.^34^, ^35^ Examples of tweets reported verbatim in our analysis are from corporate accounts or public figures for whom there is a reasonable expectation of publicity,^36^ or we have obtained explicit permission to quote the tweet. Examples for which it has not been possible to obtain permission have been paraphrased.

## 3 RESULTS

**Figure 1:**
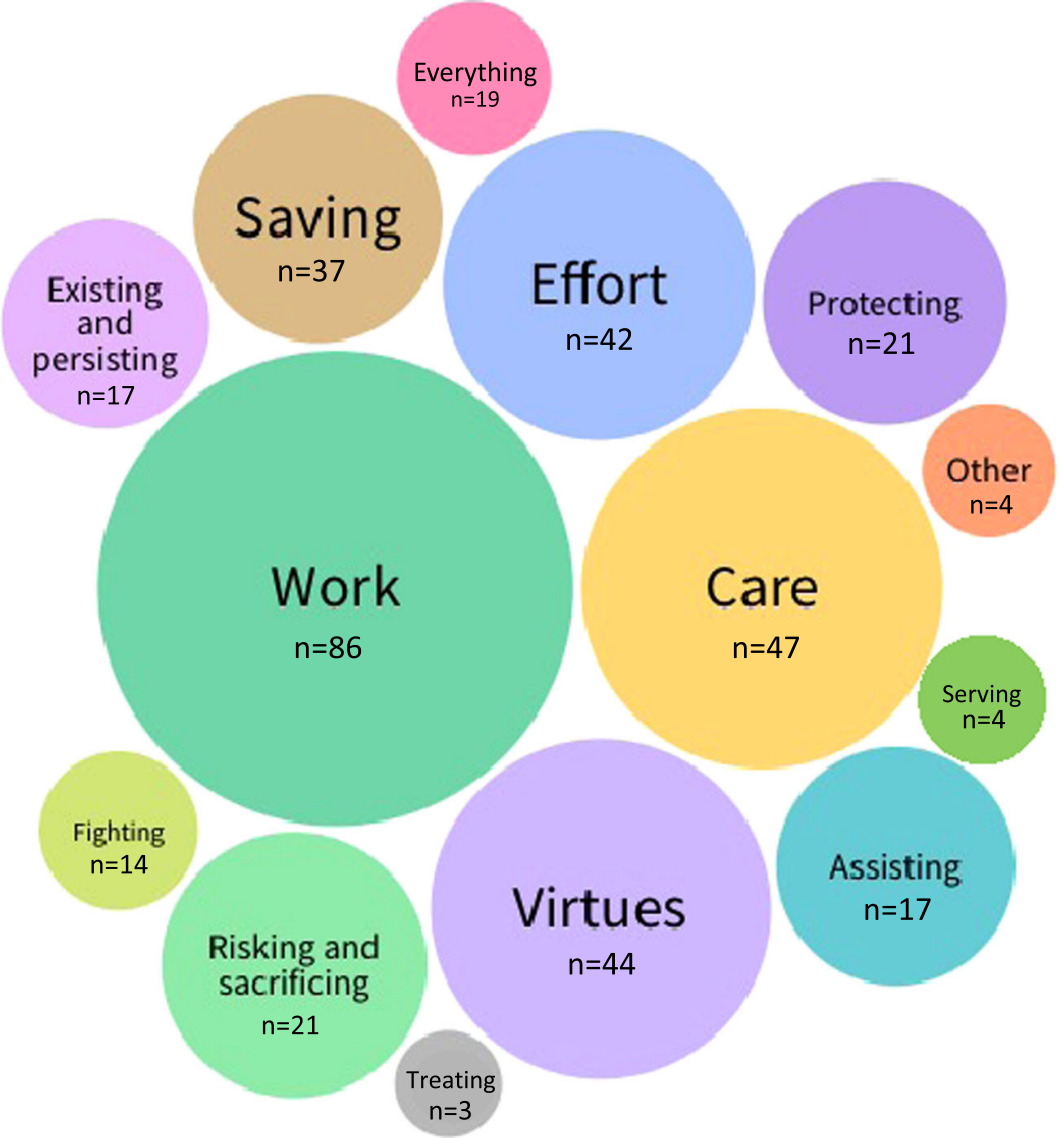
What the National Health Service was thanked for in tweets of gratitude (themes receiving >1 mention)

Most of the expressions of thanks to the NHS in our data set were ‘behabitives’, in that they enacted the social behaviour of thanking by their very expression.^37^ These expressions were often implicated in a variety of other functions, of which the most prominent were sharing news, describing care experiences, giving instructions or making requests and commenting, critiquing and criticizing. Gratitude was also harnessed to narratives of generosity through offering or receiving benefits (such as donations of goods and discounts) and fundraising as a material form of gratitude. Figure 1 shows a thematic analysis of the free coding of text in tweets that were specific about what the NHS was being thanked for. We have aggregated personal attributes for which people were thanked under ‘virtues’: these were dominated by dedication, selflessness, kindness and bravery, but commitment, courage, generosity, positivity and compassion were also mentioned.

A frequency analysis over time (Figure S1) shows that the number of tweets expressing gratitude to the NHS ramped up in the days preceding lockdown. For the next 5 weeks, a cyclical pattern of peaks is evident, showing that the social movement campaign, clap‐for‐carers, on Thursday evenings served as a potent attractor for tweets of gratitude over this period. Although there was no one turning point at which gratitude to the NHS became less visible in our data set, by the end of April, criticisms of clap‐for‐carers were beginning to take effect and the event started to lose traction. This is consistent with the findings of McKay et al.,^38^ who, in their analysis of tweets associated with the NHS and COVID during the first lockdown, found a decrease in engagement after the first month, which they attribute to lockdown fatigue and the effects on tweeting habits of the limiting experience of staying at home.

### 3.1 The clap‐for‐carers effect

Clap‐for‐carers, or more properly, Clap‐for‐our‐carers, was a UK‐wide campaign that encouraged people to take to their doorsteps, balconies and windows to give a round of applause to NHS workers and other key workers every Thursday night at 8 pm. It originated from an Instagram post from Dutch Londoner, Annemarie Plas. She was inspired by videos posted on social media of hospital healthcare workers being applauded in Italy in mid‐March 2020, a practice that spread quickly around Europe.^39^ By the end of March, the applause had become a worldwide phenomenon. After the first event in the United Kingdom on 26 March garnered press and celebrity endorsement, a professional communications agency worked with Plas on a website and visual identity for the campaign that persisted for the 10 weeks of the first lockdown in the United Kingdom. Official figures do not exist for how many people took part in clap‐for‐carers, but it was put at ‘millions’^40^ and a YouGov poll of 1664 adults in June 2020 found that 69% of respondents said they had taken part at least once.^41^

**Figure 2:**
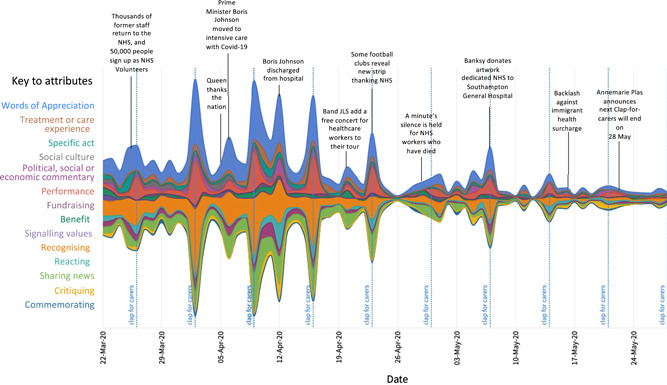
Evolution of types of tweets of gratitude during the first UK lockdown, annotated with relevant events. The size of each individual stream shape is proportional to the occurrence of attributes of tweets

Figure 2 shows a streamgraph of our coding of tweets over time. Tweets associated with clap‐for‐carers tended to include performances, with videos of applause often shared in these tweets. Tweeters also used it as an opportunity for words of appreciation directed to the NHS and key workers—numbers of tweets coded for ‘recognising’, ‘performance’ and ‘words of appreciation’ peaked on Thursdays throughout the study period.

Towards the end of April, clap‐for‐carers started to lose its potency as the initiative became more divisive, exemplified by the tweet:

Of course I am still grateful to the NHS workers themselves but clapping is beginning to feel inappropriate when what they need is PPE and a properly funded health service (30 April, paraphrased).

Participation in clap‐for‐carers featured in two of the three most ‘liked’ tweets in our corpus, both from 26 March 2020: One shared a video of the Duke and Duchess of Cambridge's children clapping and the second was of the Prime Minister and Chancellor of the Exchequer clapping outside 10 Downing Street. However, the tweet that gained the most traction (>300,000 likes, >40,000 retweets) was posted on 6 April and showed an 84‐year‐old man being wheeled out of intensive care to the applause of hospital staff, having, against the odds, recovered from COVID‐19. This role reversal in which healthcare workers—the original audience for the applause—reciprocated the clapping often featured on social media posts. These tweets were either in the context of scenes outside hospitals in which healthcare workers participated in clap‐for‐carers or staff were shown lining the corridors to applaud when patients were discharged from intensive care units.

Only the hospitalisation of UK Prime Minister Boris Johnson for treatment for COVID‐19 and his discharge from hospital rivalled clap‐for‐carers as an inducement to tweet about gratitude to the NHS. In reaction, there was a concomitant increase in tweets that harnessed thanks to commentary about pay and conditions for healthcare workers, for example:

It would be wonderful to see Boris Johnson turn his gratitude into influencing vastly improved conditions and wages for NHS staff and carers (12 April, paraphrased).

This focus on the nature of work (and, by extension, ‘doing’ in phrases like ‘all you are doing’ that is included in the category ‘effort’ in Figure 1), how it was characterized and in what ways it was worthy of gratitude featured strongly throughout our corpus.

### 3.2 The idealisation of work

Over a quarter of tweets thanking the NHS implicated work in their appreciation. The most often used qualifier was ‘hard’, but references were often made to time: ‘round the clock’, ‘24/7’, ‘day and night’. The adverb ‘tirelessly’ was most often associated with ‘working’. A typical example was:

We want to thank every person who's working tirelessly to keep this country healthy. We're so lucky to have the NHS and want you to know how grateful we are for your selfless hard work during this terrible time. We're #StayingAtHome for you and the incredible work you're doing (3 April, @jlsofficial).

Other frequently used qualifiers to describe work included ‘amazing’, ‘fantastic’, ‘great’, ‘incredible’ and ‘magnificent’.

There is pronounced asymmetry—perhaps even an irony—between the characterization of work in thankful expressions and the nature of the work being thanked for: Someone can be praised for ‘doing fantastic work’, while the rationale for thanking is predicated on an acknowledgement that the work was not fantastic to do. Similarly, describing NHS staff as working ‘tirelessly’ is contradicted by the welter of narratives of strain that emphasized fatigue and exhaustion. In common with McKay et al.,^38^ we were struck by the powerful disconnect between the symbolic and the tangible that emerged in tweets relating to the pandemic. They found that symbolism centred on the language and performance of valorisation was undercut by escalating hospitalisations and deaths. This disconnect is especially evident in our data set in the ways in which ‘saving’ was referred to as a reason to thank the NHS.

### 3.3 Saving the NHS and saved by the NHS: Mutually constructed fragility

Of the 482 instances that specified what the NHS was being thanked for, 37 referred to it as saving lives and 19 to the NHS ‘keeping us safe’. Although some referred to specific treatment experiences in which ‘saving life’ was justified, most of these references were generalized:

Thank you to everyone in the NHS for tackling coronavirus and risking their lives to keep us safe (25 March, paraphrased).

The concept of the NHS keeping people safe, like ‘working tirelessly’, is difficult to square with the reality. In the early stages of the pandemic, the NHS was powerless to keep people safe. The construction of the NHS as keeping us safe contrasts with the—literal and positional—central injunction of the UK government's three‐part slogan at the beginning of lockdown: ‘Stay at home. Protect the NHS. Save lives’. This phrase, found to be very effective in mobilizing public affect,^42^ signals the vulnerability of the NHS and links the saving of lives to the public staying at home. However, the representation of the NHS in tweets of gratitude enact an assumption reversal in which the NHS is construed as protecting and saving.

### 3.4 Caring made visible

In her wide‐ranging exploration of care published at the start of the pandemic, Bunting^43^ describes care as the ‘invisible heart’ and calls for greater acknowledgement in terms of recognition, funding, respect and value. The provision of care extends to a much wider context than that provided in the NHS to which our analysis specifically speaks. However, the prominence of care as an object for people's thanks suggests that care became more visible, and better appreciated, in pandemic discourse. The word ‘care’, unlike ‘work’, connotes a relationship, reinforced by the verbs with which it was often accompanied in tweeter's phrases: ‘giving care’ and ‘taking care’. Gratitude for care tended to be more specific than those for ‘work’ or ‘saving’, with about half referring to treatment experiences of named patients. The prominence of narratives of care was also apparent in our analysis of those to whom gratitude was addressed. Carers were the third most‐mentioned thanked category, after workers and staff. The word ‘carers’ in ‘clap‐for‐our‐carers’ was probably chosen for reasons of alliteration, but, given the early‐stage success of the campaign, it may have contributed to raising the profile of *all* carers. An outcome may be that the social reimagining of care work done by those exposed to risk and precarity, as called for by Rossiter and Godderis,^44^ becomes more likely in a postpandemic world.

### 3.5 Meaningless or meaningful?

Sorace has argued that gratitude is the ‘ideology of sovereignty in a crisis’, too easily slipping from the recognition of individuals to an acceptance of the systems that reproduce their exploitation.^45^ When gratitude was suspected as being used as a substitute currency—supposed to compensate for low pay and unsafe working conditions, or to offset policies deemed exploitative—it was reacted to, unsurprisingly, with suspicion and resentment:

I wasn't bowled over with gratitude with the clapping for the NHS. Great for morale but does not give us equipment or protective gear. Nice gesture but gestures don't translate to treatment (27 March, paraphrased).

The government is determined to depoliticize this crisis. Badges for carers it fails to protect, handprints for refugees it fails to fund, heartfelt thanks to the NHS staff it fails to equip. No. The UK's disaster is not an act of God, but of epic criminal mismanagement (20 April, @jonlis1 with permission).

An alternative construction of gratitude, though, most prevalent in our data set, is that expressions of thanks elicited reciprocal gratitude and brought moments of pleasure amidst the awfulness of the pandemic:

Coming out of work tonight and there's a huge sign at the entrance of the hospital that members of the public have made saying ‘thank you NHS workers’. It's little things like that that make you smile at the end of a 12 h shift (Smiling face emoji) (23 March, paraphrased).

On way home from work this evening called into a garage, the young man serving me noticed my lanyard and asked if I worked for the NHS. I said yes, there's a free tea or coffee there for you he said. Such a small act of kindness at the end of a long day spoke volumes. Thanks (24 March, @eamroulston with permission).

My wife and I are #NHS consultant radiologists preparing for surge in #COVID19, with 3 small children. Close to tears from this unsolicited act of kindness from our wonderful neighbour Emma, who left this on our doorstep. Thank you! #ProtectOurNHS #StayHomeSaveLives #clapforNHS (17 April, @hudson_benjamin with permission).

In this instance, gratitude is not ineffectual. Neither was it unimportant for those proffering their gratitude, many of whom found it to be a ‘feel good’ moment of solidarity:

#ClapForCarers was the moment we all needed. NHS workers are the best of us. Thank you everyone (26 March, paraphrased).

Extraordinary support and togetherness across the country for our brave and brilliant NHS doctors, nurses and carers. Brings a tear to the eye. Wonderful. Thank you (26 March, @GaryLinekar).

I think one reason why people really go for the clapping—apart obviously from wishing to show gratitude to the NHS—is that it's our one chance now to do anything at all communal. And humans do actually need communality (9 April, @baddiel).

Emotions voiced by tweeters that were often allied to expressing gratitude, particularly in association with clap‐for‐carers, were pride, love and hope. In our data set, about one in six tweets invoked solidarity and ‘togetherness’ as a value that they appreciated.

## 4 DISCUSSION

This study set out to explore gratitude expressed in tweets to the NHS during the early part of the pandemic. We adopted a methodological approach that moved away from the dominant sociocognitive model for investigating gratitude to one that is more discursive. We followed the stance of those exploring other situated social actions, such as apologies,^46^ in treating variation and contradiction as prime analytic resources. Online incivility and harassment in communication is often associated with Twitter,^47^, ^48^ but gracious communication receives barely any research attention. While gratitude was linked to cogent criticism, sarcasm, self‐aggrandizement, parody, ‘virtue signalling’ and hypocrisy in some tweets, the overwhelming majority of tweets in our data set highlighted a different, more civil, side of Twitter: One in which gratitude was associated with recognition, appreciation, valorization, congratulations, respect, compassion, generosity, humility and enthusiasm.

A notable finding was that a nationwide, communal event—clap‐for‐carers—served as a nexus for thanking activities on social media, particularly in its first few weeks. Our analysis shows that meanings imputed to the acts of gratitude were highly mobile over our study period. Gratitude became the subject of competing and conflicting notions over what ought to be the focus of press and public attention, notions that were proxies for ideological battles over roles and responsibilities. In tweets about what constituted appropriate gratitude, displays of appreciation were characterized as being incommensurable with failures of responsibility. This applied both to tweets addressed to politicians (‘if you were truly grateful to the NHS you would ensure that healthcare workers had PPE’) and to the public (‘it's very irresponsible to clap for the NHS on Thursday nights and then fail to follow the advice on social distancing’). The clap‐for‐carers case shows that the initial unity of purpose for the event lost coherence as it became ritualized and attracted criticism as being out of step with the exigencies of the pandemic. In the later stages of lockdown, gratitude was construed as misplaced and, in some cases, as offensive. It induced guilt in NHS workers, who felt they were not able to do enough to merit the public adulation or felt pressured to act in ways that went beyond what could reasonably be expected.^49^ Greenberg et al.^50^ have drawn parallels with the NHS during the pandemic and the military to explicate the threat of ‘moral injury’—a term describing psychological distress that results from challenges to one's moral code—applicable to healthcare workers having faced impossible decisions about the allocation of scarce resources to equally needy patients. Moral injury may have been exacerbated by the rhetoric of valorization.

Our analysis of repeated instances of thanking in tweets in the specific context of the COVID‐19 pandemic in the United Kingdom revealed common repertoires of gratitude circulating in the public discourse surrounding the NHS. Many of the constructions of gratitude in our data set could be described as ‘wishful thinking’ in that they cast the NHS as indefatigable and praised it for achieving the impossible during the early part of the pandemic: Keeping us safe. Further work is warranted on whether thanking routines are intrinsically hyperbolic, perhaps as an intensification strategy, and/or if concepts circulating in public discourse (like ‘tired’ and ‘save’) have their cognates more readily incorporated into thanking expressions, irrespective of descriptive accuracy. The conceptual mapping of the sociolinguistics of thanking expressions merits further research.

### 4.1 Implications

Our study supports the contention by Shaw^51^ that gratitude is implicated in assurances of ‘mattering’ that contribute to a moral community. Benefits that stimulate gratitude convey the notion that others care about us and that we are worthy of their care. There is an evidential base for gratitude being linked to social, emotional and psychological well‐being.^52^ In the face of a concomitant mental health pandemic, the denigration of acts of gratitude, on social and in mainstream media, may discourage people who could potentially benefit from the well‐being effects of practising gratitude. Previous research has concluded that people value opportunities to express their thanks.^4^ A recent House of Commons report highlighting the impact of workforce burnout identified lack of recognition as a significant contributor to feelings of ‘abandonment’ from sectors, like social care and pharmacy, that felt excluded from the public recognition being afforded the NHS in the early part of the pandemic.^53^ As we ‘build back better’, attention needs to be paid to spaces and places that accommodate gratitude—not only in healthcare but also in society in general. This is not to say that expressions of gratitude should be immune to criticism, but people's anticipation of accusations of inauthenticity and virtue signalling may discourage thanking activities that, if enacted, could make a real difference to motivation and morale.

Our study also has implications for how the pandemic is remembered in popular culture and commemorated. Clapping has already become a shorthand for social appreciation, although sometimes with ironic overtones. We need look only to the way in which sentiments like ‘Blitz spirit’ still influence people's strategies for coping in times of crisis^54^ to realize that the way the pandemic is commemorated will influence how we respond to future crises. Given how contentious thanking the NHS became during the pandemic, the ways in which gratitude to healthcare workers is incorporated into commemorative acts and material culture should be the subject of extensive consultation to maximize its chances of striking the appropriate tone.

### 4.2 Strengths and limitations

An innovative aspect of this study is that we have developed a typology for thanking expressions that may be applicable to categorizing gratitude in other contexts. This study benefited from a robust approach to data collection and analysis. Tweets were considered as a whole—including images and videos—which are not usually captured by data‐scraping methods. The consensual approach to coding, while time consuming, allowed for a reflexive attitude to our data. We do not claim, however, that tweets constitute the ‘naturally occurring talk’ preferred by those using a discursive psychology methodology. Although they are ‘natural’ in that they are not intentionally solicited by a researcher, the search‐and‐retrieval methods necessary to assemble a data set, and the opacity of Twitter's proprietary search algorithms, make data retrieval analogous to elicitation.

Acts of true creativity in thanking practices are likely to employ semantics that elude search strings even when those acts are highly culturally salient. An example is that the discourse surrounding Captain Tom's^55^ extraordinary fundraising activities for NHS Charities did not feature strongly in our data set in spite of being a dominant narrative that featured an outpouring of gratitude during the first lockdown. Thanking exchanges took place mainly between Captain Tom and donors to his campaign, with the NHS being invoked only occasionally in tweets that fulfilled our inclusion criteria. By focusing on ‘micronarratives’, some of the ‘macronarratives’ may be underrepresented, both because of our restricted search terms and by the purposive selection of attention‐garnering tweets rather than relying on a random sample. As Venditti et al.^30^ have pointed out, social media use is driven by more than the spontaneous practices of users—it includes strategized activities, including ‘liking’, that are determined by the specific architecture of the platform. Insights into the pragmatics of user interactions are not available to researchers examining content, and care needs to be taken not to equate ‘liked’ tweets with public approval beyond the context of Twitter.

## 5 CONCLUSION

We have presented a framework for analysing gratitude expressed on social media that we applied to attention‐attracting tweets that engaged with gratitude to the NHS during the first lockdown of the COVID‐19 pandemic in the United Kingdom. Thanking practices and attitudes to gratitude were dynamic and responsive to events. Gratitude was both *performed* and *critiqued* on Twitter, affording valuable insights into its discursive function and social value. Our quantitative data indicated distinct patterns of activity, complementing our qualitative analysis that investigated the purpose and content of expressions of gratitude. The ambivalence surrounding gratitude revealed in our study does not render it unhelpful as a sociological construct. On the contrary, it highlights the volatility of emotional ritualized social performances and how susceptible these are to context. Our study shows that gratitude has figured as a prominent, if contentious, social value, catalysing debates about social behaviours and prompting a reappraisal of the risks and rewards of healthcare and social care work.

## CONFLICT OF INTERESTS

The authors declare no conflicts of interest.

## AUTHOR CONTRIBUTIONS

All authors were involved in devising the method, interpreting the data and editing and revising the manuscript. In addition, Giskin Day conceptualized the project, compiled the data set, coded data and drafted the original manuscript, Glenn Robert and Kathleen Leedham‐Green coded data and Anne Marie Rafferty audited the coding.

## Supporting information

Supporting information.

## Data Availability

Data on which the analysis is based are publicly published on Twitter. Data elicitation and anonymization methods have been described in the manuscript. Because this data set is dynamic, it may not be possible to exactly replicate the data set. The data set that supports the findings of this study is available upon reasonable request from the corresponding author.

## References

[1] Steinert S . Corona and value change: the role of social media and emotional contagion. Ethics Inf Technol. 2020:1‐10. 10.1007/s10676-020-09545-z PMC7372742 32837288

[2] Wood H , Skeggs B . Clap for carers? From care gratitude to care justice. Eur J Cult Stud. 2020;23(4):641‐647. 10.1177/1367549420928362

[3] Chatzidakis A , Segal L . Do we really “All Care Now”? Time to expand our caring imagination. *Red Pepper*. April 2020.

[4] Day G . Enhancing relational care through expressions of gratitude: insights from a historical case study of almoner–patient correspondence. Med Humanit. 2020;46(13):288‐298. 10.1136/medhum-2019-011679 31586010 PMC7476306

[5] Gillespie A , Reader TW . Identifying and encouraging high‐quality healthcare: an analysis of the content and aims of patient letters of compliment. BMJ Qual Saf. 2020;30:484‐492. 10.1136/bmjqs-2019-010077 PMC8142452 32641354

[6] van Diepen C , Wolf A . “Care is Not Care if it Isn't Person‐Centred”: a content analysis of how person‐centred care is expressed on Twitter. Health Expect. 2021;24:1‐8. 10.1111/hex.13199 PMC8077091 33506570

[7] Ma LK , Tunney RJ , Ferguson E . Does gratitude enhance prosociality?: a meta‐analytic review. Psychol Bull. 2017;143(6):601‐635. 10.1037/bul0000103 28406659

[8] Syropoulos S , Markowitz EM . Prosocial responses to COVID‐19: examining the role of gratitude, fairness and legacy motives. Pers Individ Dif. 2021;171:110488. 10.1016/j.paid.2020.110488 35502308 PMC9045807

[9] Tong EMW , Oh VYS . Gratitude and adaptive coping among Chinese Singaporeans during the beginning of the COVID‐19 pandemic. Front Psychiatry. 2021;11:1‐8. 10.3389/fpsyt.2020.628937 PMC7870711 33574774

[10] Dennis A , Ogden J , Hepper EG . Evaluating the impact of a time orientation intervention on well‐being during the COVID‐19 lockdown: past, present or future? J Posit Psychol. 2020;00(00):1‐11. 10.1080/17439760.2020.1858335

[11] Yale . The Science of Well‐Being. Coursera. 2020. Accessed July 25, 2020. https://www.coursera.org/learn/the-science-of-well-being

[12] Fredrickson BL . Gratitude, like other positive emotions, broadens and builds. In: Emmons RA , McCullough ME , eds. The Psychology of Gratitude. Oxford University Press; 2004:145‐165.

[13] Day G , Robert G , Rafferty AM . Gratitude in health care: a meta‐narrative review. Qual Health Res. 2020;30(14):2303‐2315. 10.1177/1049732320951145 32924863 PMC7649920

[14] Yoshimura SM , Berzins K . Grateful experiences and expressions: the role of gratitude expressions in the link between gratitude experiences and well‐being. Rev Commun. 2017;17(2):106‐118. 10.1080/15358593.2017.1293836

[15] Hurst TM . The discursive construction of superintendent statesmanship on Twitter. Educ Policy Anal Arch. 2017;25:29. 10.14507/epaa.25.2300

[16] Rasmussen J . ‘Should Each of Us Take Over the Role as Watcher?’ Attitudes on Twitter towards the 2014 Norwegian Terror Alert. J Multicult Discourses. 2015;10(2):197‐213. 10.1080/17447143.2015.1042882

[17] Wiggins S . Discursive Psychology: Theory, Method and Applications. SAGE Publications; 2017. 10.4324/9780429356032-4

[18] Veen M , Gremmen B , te Molder H , van Woerkum C . Emergent technologies against the background of everyday life: discursive psychology as a technology assessment tool. Public Underst Sci. 2011;20(6):810‐825. 10.1177/0963662510364202 22397087

[19] Edwards D . Moaning, whinging and laughing: the subjective side of complaints. Discourse Stud. 2005;7(1):5‐29. 10.1177/1461445605048765

[20] Barrett LF . How Emotions Are Made. Pan Macmillan; 2017.

[21] Kleinberg B , van der Vegt I , Mozes M . Measuring emotions in the COVID‐19 real world worry dataset. arXiv:2004.04225v2 [cs.CL]. 2020.

[22] Mitchell L , Frank MR , Harris KD , Dodds PS , Danforth CM . The geography of happiness: connecting Twitter sentiment and expression, demographics, and objective characteristics of place. PLoS One. 2013;8(5):64417. 10.1371/journal.pone.0064417 PMC3667195 23734200

[23] Jensen EA . Putting the methodological brakes on claims to measure national happiness through Twitter: methodological limitations in social media analytics. PLoS One. 2017;12(9):1‐7. 10.1371/journal.pone.0180080 PMC5589095 28880882

[24] Oxford English Dictionary Online . Gratitude; 2019. Oxford University Press.

[25] Carr D , ed. Perspectives on Gratitude: An Interdisciplinary Approach. Routledge; 2016.

[26] Lambert NM , Graham SM , Fincham FD . A prototype analysis of gratitude: varieties of gratitude experiences. Personal Soc Psychol Bull. 2009;35(9):1193‐1207. 10.1177/0146167209338071 19581434

[27] Morgan B , Gulliford L , Kristjánsson K . Gratitude in the UK: a new prototype analysis and a cross‐cultural comparison. J Posit Psychol. 2014;9:281‐294. 10.1080/17439760.2014.898321

[28] Twitter . About search rules and restrictions; 2020. Accessed July 31, 2020. https://help.twitter.com/en/rules-and-policies/twitter-search-policies

[29] Taylor C . The “Like” Doesn't Mean What You Think It Means. Mashable; 2019. Accessed July 28, 2020. https://me.mashable.com/tech/5627/the-like-doesnt-mean-what-you-think-it-means

[30] Venditti S , Piredda F , Mattana W . Micronarratives as the form of contemporary communication. Des J. 2017;20(suppl 1):S273‐S282. 10.1080/14606925.2017.1352804

[31] Haugh M . Im/politeness, social practice and the participation order. J Pragmat. 2013;58:52‐72. 10.1016/j.pragma.2013.07.003

[32] Hill CE , Thompson BJ , Hess SA , Knox S , Williams EN , Ladany N . Consensual qualitative research: an update. J Couns Psychol. 2005;52(2):196‐205. 10.1037/0022-0167.52.2.196

[33] Spangler PT , Liu J , Hill CE . Consensual qualitative research for simple qualitative data: an introduction to CQR‐M. In: Hill CE , ed. Consensual Qualitative Research: A Practical Resource for Investigating Social Science Phenomena. American Psychological Association; 2012:269‐283.

[34] Franzke AS , Bechmann A , Zimmer M , Ess CM . Association of Internet Researchers Internet Research: Ethical Guidelines 3.0; 2020.

[35] Williams ML , Burnap P , Sloan L . Towards an ethical framework for publishing Twitter data in social research: taking into account users' views, online context and algorithmic estimation. Sociology. 2017;51(6):1149‐1168. 10.1177/0038038517708140 29276313 PMC5718335

[36] National Committee for Research Ethics in the Social Sciences and Humanities. *A Guide to Internet Research Ethics*; 2019.

[37] Austin JL . How to Do Things with Words: The William James Lectures Delivered at Harvard University in 1955. Oxford University Press; 1965.

[38] McKay K , Wayland S , Ferguson D , Petty J , Kennedy E . “At Least until the Second Wave Comes…”: a Twitter analysis of the NHS and COVID‐19 between March and June 2020. Int J Environ Res Public Health. 2021;18(8):3943. 10.3390/ijerph18083943 33918586 PMC8069751

[39] Plas AM clapforourcarers.com. Instagram; 2021. Accessed April 24, 2021. https://www.instagram.com/p/B-AJkisAhSF/

[40] Coronavirus: Millions Take Part in Weekly “Clap for Carers” Tribute . BBC News; 2020. Accessed April 24, 2021. https://www.bbc.co.uk/news/av/uk-52402181

[41] Abraham T . Third of Britons Think Clap for Carers has been Politicised. *YouGov*; 2020.

[42] Jackson J , Posch C , Bradford B , Hobson Z , Kyprianides A , Yesberg J . The Lockdown and Social Norms: Why the UK is Complying by Consent Rather than Compulsion. British Politics and Policy Blog at LSE; 2020. Accessed August 28, 2020. https://blogs.lse.ac.uk/politicsandpolicy/lockdown-social-norms/

[43] Bunting M . Labours of Love: The Crisis of Care. Granta; 2020.

[44] Rossiter K , Godderis R . Essentially invisible: risk and personal support workers in the time of COVID‐19. Sociol Health Illn. 2020;42(8):e25‐e31. 10.1111/1467-9566.13203 33156530

[45] Sorace C . Gratitude: The Ideology of Sovereignty in Crisis. *Made in China Journal*. 2020. Accessed July 17, 2020. https://madeinchinajournal.com//05/18/gratitude-the-ideology-of-sovereignty-in-crisis/

[46] Augoustinos M , Lecouteur A , Fogarty K . Apologising‐in‐action: on saying ‘Sorry’ to Indigenous Australians. In: Hepburn A , Wiggins S , eds. Discursive Research in Practice: New Approaches to Psychology and Interaction. Cambridge University Press; 2007:88‐103. 10.1017/CBO9780511611216.005

[47] Maity SK , Chakraborty A , Goyal P , Mukherjee A . Opinion conflicts: an effective route to detect incivility in Twitter. Proc ACM Human‐Computer Interact. 2018;2(CSCW):1‐27. 10.1145/3274386

[48] Theocharis Y , Barberá P , Fazekas Z , Popa SA . The dynamics of political incivility on Twitter. SAGE Open. 2020;10(2):371. 10.1177/2158244020919447

[49] Cox CL . “Healthcare Heroes”: problems with media focus on heroism from healthcare workers during the COVID‐19 pandemic. J Med Ethics. 2020;46(8):510‐513. 10.1136/medethics-2020-106398 32546658 PMC7316119

[50] Greenberg N , Docherty M , Gnanapragasam S , Wessely S . Managing mental health challenges faced by healthcare workers during COVID‐19 pandemic. BMJ. 2020;368:1‐4. 10.1136/bmj.m1211 32217624

[51] Shaw J . Gratitude, self‐assessment, and moral community. J Value Inq. 2013;47:407‐423. 10.1007/s10790-013-9396-7

[52] Jans‐Beken L , Jacobs N , Janssens M , et al. Gratitude and health: an updated review. J Posit Psychol. 2019;15:1‐40. 10.1080/17439760.2019.1651888

[53] House of Commons Health and Social Care Committee . *Workforce Burnout and Resilience in the NHS and Social Care*. London; 2021.

[54] Jones E . The psychology of protecting the UK public against external threat: COVID‐19 and the Blitz compared. Lancet Psychiatry. 2020;0366(20):1‐6. 10.1016/S2215-0366(20)30342-4 PMC7834303 32861267

[55] Captain Tom Moore: NHS fundraiser “lifted lockdown blues. *BBC News*; 2020. https://www.bbc.co.uk/news/uk-england-beds-bucks-herts-53178431?intlink_from_url=https://www.bbc.co.uk/news/topics/ckr483q4dwgt/captain-tom-moore%26link_location=live-reporting-story

