# Supplementary material for: An outbreak of appreciation: A discursive analysis of tweets of gratitude expressed to the National Health Service at the outset of the COVID‐19 pandemic
Source: Health Expect. 2021 Sep 20;25(1):149–62. doi: 10.1111/hex.13359 (PMC8652934; doi:10.1111/hex.13359)

Supplemental Figure 1. Number of tweets meeting inclusion criteria retrieved by Twitter search between 1 March and 21 June 2020

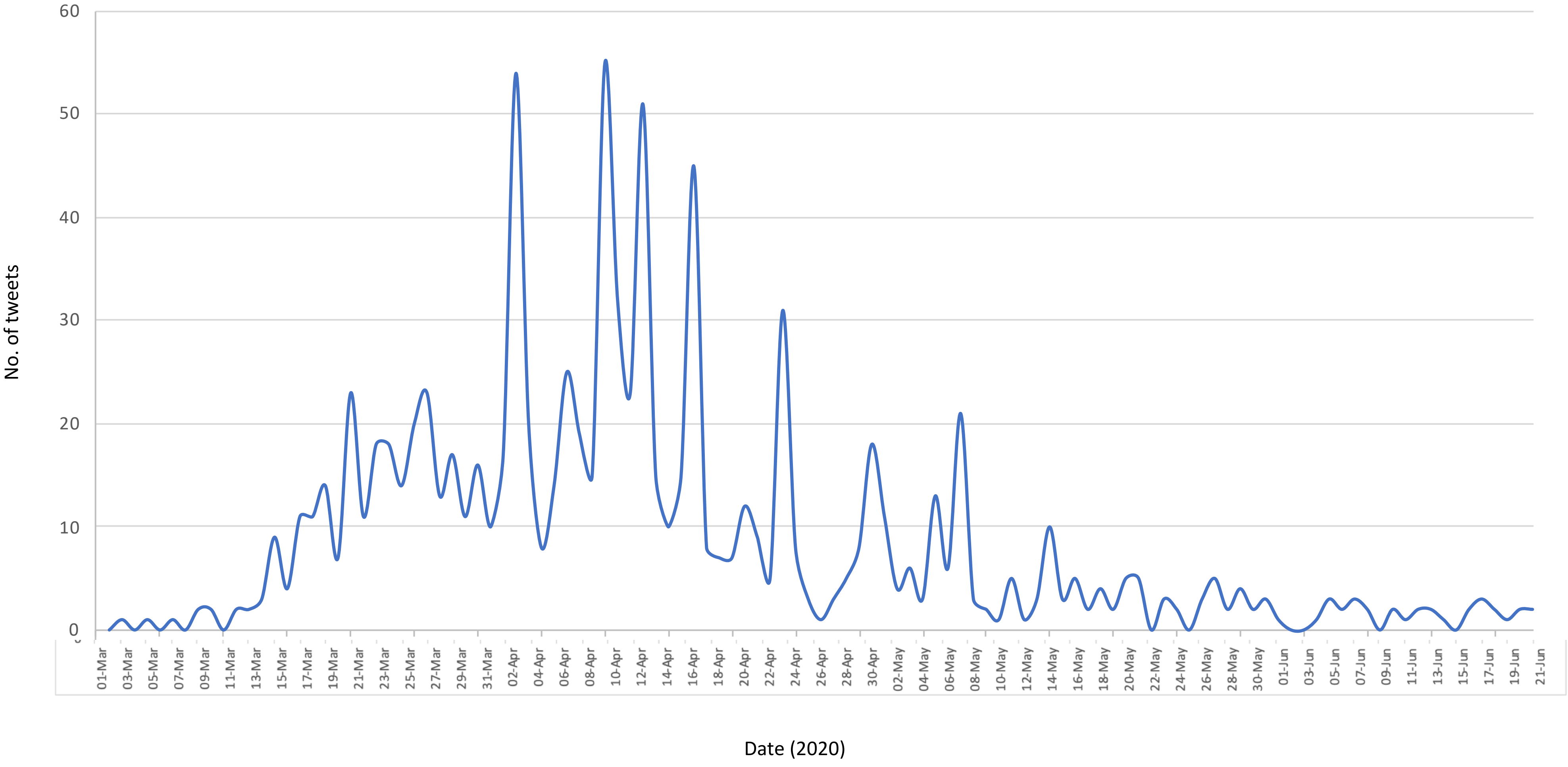

Supplement: Supplementary file 1 — Supporting information. [file HEX-25-149-s001.pdf]
